# Clinical Communication Skills Training in Dental Medical Education: The COVID-19 Pandemic Challenge

**DOI:** 10.3390/healthcare8040429

**Published:** 2020-10-25

**Authors:** Henrique Salgado, Ivone Castro-Vale

**Affiliations:** 1 Psychiatry and Mental Health Clinic, University Hospital Centre of São João, Al. Prof. Hernâni Monteiro, 4200-319 Porto, Portugal; p.henrik.s@gmail.com; 2 Medical Psychology Unit, Department of Clinical Neurosciences and Mental Health, Faculty of Medicine, University of Porto, Al. Prof. Hernâni Monteiro, 4200-319 Porto, Portugal; 3 Faculty of Dental Medicine, University of Porto, Rua Dr. Manuel Pereira da Silva, 4200-393 Porto, Portugal; 4 i3S-Institute for Research and Innovation in Health, Rua Alfredo Allen, 208, 4200-135 Porto, Portugal

**Keywords:** dental education, medical education, clinical communication skills, COVID-19 pandemic, online teaching, patient-centred care, clinical skills training

## Abstract

It is very important for healthcare professionals to have good clinical communication skills, especially dentists. Patient-centred care results in patient satisfaction, better outcomes, and less complaints from dental patients. Due to the onset of the COVID-19 pandemic, the clinical communications skills programme of the pre-graduate course in dental medicine at the University of Porto had to be transformed to an online format. Based on their role as faculty, the authors aimed to recount their initial reflections and concerns within this perspective, and to share how they adapted to the new reality of teaching clinical communication skills online, as well as the conclusions of our experience, in the hope that this will help others who may have to go through a similar process. The authors acknowledged that the students achieved the pre-established goals of the clinical communications skills programme through the new online format.

## 1. Introduction

Clinical communication skills are an essential component of a doctor’s training, both at the pre-clinical stage, and in further education medical programmes [1]. Teaching clinical communication skills to future dentists is extremely important, as it has been shown that good clinical communication increases patient satisfaction, is associated with better outcomes, and decreases the level of patients’ complaints [2,3,4]. Many medical schools have structured communication skills courses in undergraduate and postgraduate medical education [5,6]. Extending communication skills training to all fields of healthcare demonstrates its increasingly-recognised importance for the clinician-patient relationship in a patient-centred model of care [6,7,8]. Dental schools of medicine are no exception [5]. Indeed, communication skills are essential for building a good relationship between dentist and patient [9], and communication skills training has been shown to improve the communication skills of dental students [5]. Clinical communication skills training should be carried out step-by-step, starting with the teaching of basic knowledge during the earlier, generally pre-clinical stages of dental medical education with theoretical and practical classes, which includes discussing didactic episodes and clinical scenarios, as well as learning by modelling and supervised role-playing with peers [5]. The next steps consist of role-playing with simulated patients, through to the final step of training with real patients. Experienced instructors have to be present throughout the process, providing feedback and promoting reactions and comments from all students [6,7,10]. The syllabus of the pre-graduate course programme of the Masters in Dental Medicine of the Faculty of Dental Medicine at the University of Porto (FMDUP) includes two course units in the second year; Psychology I, in the first semester; and Psychology II, in the second semester, during which clinical communication skills are taught. As the subject is lectured during the pre-clinical year, role-playing is only carried out amongst peers. The teaching methodology is similar to that described elsewhere during the initial phase [11], where the focus is on teaching basic communication skills in the first semester, and specific communication skills in the second semester, such us responding to strong emotions, breaking bad news, the motivational interview, and communication with children and adolescents, among other subjects [12].

Teaching clinical communication skills to future dentists cannot come to a halt on account of the COVID-19 pandemic [13]. It is from this perspective that the authors, all of whom are members of faculty of the pre-clinical communication skills training programme at the FMDUP, aim to share their experience in transforming this in-person programme into an online one, which is completely taught at a distance, describing the advantages and disadvantages, of this change and the lessons that can be learnt, which can be useful to other teachers, as the pandemic is far from its end [14]. This perspective paper was approved by the Ethics Committee of our University (Comissão de Ética para a Saúde do Centro Hospitalar São João/Faculdade de Medicina da Universidade do Porto, approval number: CE-OP-49-2020).

## 2. Discussion

The onset of the COVID-19 pandemic has led to an unprecedented impact on the general Portuguese community, which found many of its activities suspended and others being redefined in accordance with governmental stay-at-home measures. Education in general was no exception, affecting students, teachers, and families alike. In particular, dental medical schools were closed, which created, among other problems, a huge challenge for teachers who had to adapt rapidly to new technologies for teaching at a distance. This closure, which took place shortly after the beginning of the second semester, forced the creation and adaptation of new teaching methodologies. Accordingly, in general, we witnessed a change from in-person classroom teaching to teaching at a distance, using online tools and platforms, with the exception of those clinical practice which treated real patients of the clinical years. Specialised groups comprising members of faculty and students were formed to design, develop, and support online teaching practices. The University of Porto was already using applications and software platforms for distance learning, although only some courses and faculty had already implemented them at the time of the onset of the COVID-19 pandemic. This change required a major adaptation of the academic community, as the University exerted pressure for all in-person classes to be replaced by online teaching, if possible. Most pre-clinical course units were relatively easily transformed from in-person classes to online classes. Whereas many of the course units managed to adapt to new methods with little difficulty (especially those which have a greater theoretical component in their syllabus), some other course units encountered more difficulties, due to a greater practical component of teaching, which was the case for the teaching of clinical communication skills in the Psychology II course unit of the Masters in Dental Medicine at the University of Porto.

As the first cases of COVID-19 were diagnosed in Portugal, everything was evolving so rapidly that we had to rapidly reflect and decide on some questions, such as: How could we replace in-person role-playing with online role-playing, as this represents the ultimate achievement of our first step of teaching clinical communication skills? Can students be taught online how to respond to strong emotions, or how to give bad news? What about non-verbal-communication? Would it be feasible to rehearse behind the lens of a computer camera? Would students be willing to engage in role-playing at all under these circumstances? What would students lose by online teaching in terms of learning clinical communication skills, where the presence of the healthcare professional has a value on its own? Would it be possible to postpone in-person classes if the pandemic crisis subsided? In addition, ethical issue also arose related to the possibility of the unauthorised video-recording of the classes, the varying conditions affecting students when being taught using real time platforms, and also whether students should be obliged to turn on their cameras during the whole of the class.

Before we even answered all of these questions, and while the number of COVID-19 cases was rapidly increasing, our weekly classes were interrupted on 17 March 2020 when the FMDUP was abruptly closed, resulting in the suspension of clinical communication skills classes. These classes were temporarily replaced by a discussion forum of clinical cases related to the topics that had been interrupted (using the Moodle Learning Management System platform, with which both students and faculty were already familiar). Simultaneously, we reflected on the various above-mentioned questions and also tried to understand the position of the students. Theoretical classes continued in the form of real-time sessions using the Zoom platform. However, when it became clear that there was a need to extend teaching at a distance throughout the rest of the semester, due to the increasing uncertainty regarding the evolution of the COVID-19 pandemic which had been declared by the WHO on March 11th [15], the methodology had to be quickly rethought. At the same time, the students demonstrated a strong interest in resuming classes online, even though they were informed that the experience would not be the same. Their motivations were related to the need to have the best possible learning of the Psychology II course unit, rather than be taught nothing at all, as the postponing of in-person classes did not seem to be realistic as time passed by. In addition, we also think that their familiarity with the Psychology I course unit of the first semester and also with the online teaching platforms constituted an important contribution for their motivations. As faculty, we considered that these motivations were very important for the success of the online methodology. After having explained, in detail, what the students’ contribution in the online classes was expected to be, we made all the necessary adaptations to compensate for the differences, and although the methodology was to change, our perception was that the quality of the content being taught did not. Accordingly, clinical communication skills classes started to be carried out online through real-time video-conference sessions (using Zoom), starting with an initial discussion regarding the topic between the member of faculty and the students, with the former stimulating the latter to ask questions. Shortly after, students were taught through the means of pre-prepared clinical situations in the form of role-playing among peers (whereby the student who was carrying out the role of the patient was given a script), which included supervision and feedback from the member of faculty, with observation and discussion of what happened by all students. As online classes progressed, the students became very receptive and easily adapted to the real-time video-conference sessions, as confirmed by the faculty’s perception and the frequent and enthusiastic participation of the students in the role-playing and the overall discussions. The system of sharing image and sound in real-time enabled an adequate speed of communication in most situations, and also permitted learning from certain aspects of non-verbal communication, such as verbal prosody and body kinesis (e.g., facial expression or gesticulation), which kept the sessions alive, even though they were online, as well as the therapeutic alliance construction process. Supervision, observation, and discussion of the practices observed were also satisfactorily achieved, with the role-playing being well-accompanied remotely by colleagues and a member of faculty, which provided interesting opportunities for debate and learning for all. These assertions were a result of both the reflections of the faculty and the students’ comments regarding the opportunities to discuss the challenges of clinical communication related to the specific subject of each class, and also of the clinical cases that were role-played. The students’feedback was always stimulated, and as they already had experience of in-person teaching during the first semester classes of basic clinical communication learning, in our opinion they were able to make a comparison between in-person and online learning, although no objective assessment was made. These inferences warrant the carrying out of objective studies resulting from this perspective to obtain more reliable conclusions.

Although undoubtedly these new technologies provided positive aspects, some of the aspects of the teaching of clinical communication skills were lost when compared with in-person role-playing. Some artificiality of the dialogue could have lead a decrease in the student’s sense of presence during the role-playing, which could possibly be impaired by the communication barriers inherent to the technology used, which could well generate a greater feeling of interpersonal distance. In addition, non-verbal communication, such as touch, is impossible when cameras are used for role-playing. The system itself (even should the ideal conditions be achieved in terms of the quality of internet, image, and sound) is not infallible, with the possibility of failures in the quality of verbal and non-verbal communication, even for short periods. Although the students were generally compliant in turning on their cameras, some were reluctant to turn them on for the whole duration of the classes, however, in general, the students were present during the whole time, and the attendance rate was higher than during the first semester. Being confined to their home probably meant that the students were more available to attend online classes. However, as they were in their homes with other family members or a roommate, this sometimes led to them turning off their cameras, and the sound also. Interestingly, the online theoretical classes, which are facultative, experienced a significantly-increased attendance level by students when compared to the period before the onset of online teaching. The increased level of attendance for both the theoretical and practical online classes warrants further study. The various aspects of online teaching were evaluated through the use of several different methodologies, including the classic multiple-choice tests and the performance achieved in the participation in both practical classes and role-playing. The multiple-choice tests were also carried out online, using the Moodle platform, with the Safe Exam Browser, which had been used before, with invigilation being carried out by using Zoom. Although teaching was carried out online, the original learning objectives were maintained, with the evaluation standards and methodology only being adapted to reflect online teaching. Importantly, at the end of the online teaching of the Psychology II course unit, the students’ grades were very similar to those achieved for the Psychology I course unit, which was taught in-person during the first semester. It should be mentioned though that the grades of the Psychology II course unit were also similar to those of the previous year, before the COVID-19 pandemic, although they are not really comparable, owing to the restructuration of the Psychology I and II course units of this year, which resulted in dedicating more time to teaching clinical communication skills.

After weighing up all the advantages and disadvantages, we have no doubt that the overall balance of the online teaching method of clinical communication skills using the latest internet technologies was quite positive. In the end, we felt that there had been a good evolution of the students’ learning of clinical communication skills, despite the difficulties inherent to the context.

As there is no forecast end to the pandemic, with a possibility of the need to return to confinement, together with students’ preferences to not resume in-person classes while the pandemic continues, even in the case of pre-clinical years [16], this online method of teaching has become a probable necessary alternative. Furthermore, the return to clinical placement may well pose a challenge to students in terms of their doubts of how to communicate with patients. These strategies can be used for those students who feel a need to improve their clinical skills and can be used with both standardised patients and real-life patients [17]. However, it is extreme likely that a part of the teaching of dental medical students during their clinical years will nowadays include tele-consultation, as this has become a generalised practice for some clinical settings, although there have been reports of students experiencing difficulties with establishing a relationship with the patient in this context. However this could be overcome by applying similar methodologies of teaching clinical communication skills as those described above, especially the adoption of role-paying among peers or with standardised patients, or even with real-life patients [18].

Ultimately, when the pandemic finally comes to an end, we believe that in-person classes are the preferred method for teaching clinical communication skills, particularly during the pre-clinical years. One important aspect of learning clinical communication skills is related to building a relationship with the patient. As the aim is to train future doctors who will treat patients in person, it is very important that students be able to practice these skills in person. Even though theoretical classes can more easily be taught online if necessary, the physical presence of the member of faculty is enriching for both the student and the member of faculty themselves, as the latter can promote more spontaneous interactions. However, as mentioned in the previous paragraph, there are various other situations where online teaching can definitely be of use.

## 3. Conclusions

The onset of the COVID-19 pandemic led to a sudden and unexpected significant change in our way of living, working, and teaching, including that of clinical communication skills to dental medical students.

Adaptation to the need to change teaching methods was rapidly implemented, and although certain aspects regarding the learning objectives may not have been achieved, we can conclude that the online teaching of clinical communication skills using applications and software platforms is possible. Indeed, as members of faculty of the clinical communications skills programme of the pre-graduate course in dental medicine, the authors perspective is that the results have been positive, and that online teaching can be continued for as long as needs be, as it seems that the COVID-19 pandemic is far from over, as has recently been announced by the World Health Organization, and it is highly likely that new periods of confinement will be necessary again. This online teaching methodology can also be justified in cases where it is impossible to implement social distancing, such as when conditions favour large class sizes and/or insufficient space is available.

Furthermore, we conclude that there is a need for more research in the form of quantitative and qualitative studies to better characterise the advantages and disadvantages of in-person and online teaching of clinical communication skills, as both appear to be of use if applied in the right context. Such future studies would provide us with more concrete information as to which method is best for each case.
